# Introduced Mammalian Predators Induce Behavioural Changes in Parental Care in an Endemic New Zealand Bird

**DOI:** 10.1371/journal.pone.0002331

**Published:** 2008-06-04

**Authors:** Melanie Massaro, Amanda Starling-Windhof, James V. Briskie, Thomas E. Martin

**Affiliations:** 1 School of Biological Sciences, University of Canterbury, Christchurch, New Zealand; 2 USGS Montana Cooperative Wildlife Research Unit, University of Montana, Missoula, Montana, United States of America; University of Kent, United Kingdom

## Abstract

The introduction of predatory mammals to oceanic islands has led to the extinction of many endemic birds. Although introduced predators should favour changes that reduce predation risk in surviving bird species, the ability of island birds to respond to such novel changes remains unstudied. We tested whether novel predation risk imposed by introduced mammalian predators has altered the parental behaviour of the endemic New Zealand bellbird (*Anthornis melanura*). We examined parental behaviour of bellbirds at three woodland sites in New Zealand that differed in predation risk: 1) a mainland site with exotic predators present (high predation risk), 2) a mainland site with exotic predators experimentally removed (low risk recently) and, 3) an off-shore island where exotic predators were never introduced (low risk always). We also compared parental behaviour of bellbirds with two closely related Tasmanian honeyeaters (*Phylidonyris* spp.) that evolved with native nest predators (high risk always). Increased nest predation risk has been postulated to favour reduced parental activity, and we tested whether island bellbirds responded to variation in predation risk. We found that females spent more time on the nest per incubating bout with increased risk of predation, a strategy that minimised activity at the nest during incubation. Parental activity during the nestling period, measured as number of feeding visits/hr, also decreased with increasing nest predation risk across sites, and was lowest among the honeyeaters in Tasmania that evolved with native predators. These results demonstrate that some island birds are able to respond to increased risk of predation by novel predators in ways that appear adaptive. We suggest that conservation efforts may be more effective if they take advantage of the ability of island birds to respond to novel predators, especially when the elimination of exotic predators is not possible.

## Introduction

The majority of bird extinctions since 1800 have occurred on islands and the main cause of these extinctions has been the introduction of exotic predators [1], [2] often in close association with drastic habitat alterations [3], [4]. The impact of introduced predators on the native avifauna of oceanic islands is particularly profound because the birds evolved largely in the absence of many predators [e.g., 5]. In continental areas, birds and predators have co-evolved over millions of years, and many behavourial and life history traits vary adaptively with risk of predation [6]–[9]. In contrast, native birds on predator-free islands appear to have lost adaptations to avoid terrestrial predators. Instead, they exhibit behaviours and life history traits (e.g. tameness, loss of flight, large size, low fecundity) that predispose them to population crises when predatory animals are introduced [10], [11], suggesting that they are evolutionarily ‘trapped’ [12], [13]. However, island birds ‘trapped’ by exotic predators are not necessarily condemned to extinction [13]. The relative risk of extinction will depend on the ability of a species to adjust behavioural traits or evolve in response to exotic predators. Yet, studies of trait changes in response to novel changes in predation risk among island birds are lacking. Here we present a detailed study of responses in island honeyeaters to variation in current and historic predation risk on New Zealand and Tasmania, Australia.

New Zealand provides a typical example of problems arising from introduction of exotic predators. Extinctions of birds on oceanic islands such as New Zealand have been directly linked to human-induced habitat destruction and the introduction of predatory mammals [2]–[4], [14]–[16]. New Zealand was first settled by Maori in ∼1300, and then by Europeans beginning in 1769. Both settlement phases were associated with the introduction of exotic mammalian predators; Maori introduced the Polynesian rat (*Rattus exulans*), while Europeans introduced the house mouse (*Mus domesticus*), two additional species of rats (*R. rattus* and *R. norvegicus*), the hedgehog (*Erinaceus europaeus*), the domestic cat (*Felis catus*), three mustelids (*Mustela erminea, M. furo* and *M. nivalis*) and the brushtail possum (*Trichosurus vulpecula*). These introductions contributed to the extinction of ∼40% of all non-marine bird species in New Zealand [3], [16], [17] and pose a major threat to the survival of the remaining avifauna [18].

In this study we tested whether an endemic New Zealand songbird, the bellbird (*Anthornis melanura*), altered its parental behaviour and life history traits in ways that might adapt it to the novel predation risk from introduced mammalian predators. In particular, increased parental activity at a nest can attract predators and increase nest predation rates [19]. Bird species adaptively differ in their rates of parental feeding visits to the nest during the nestling period related to risk of predation [8], [9], [20], [21]. Birds can also achieve lower activity during incubation by reducing the number of visits per unit time and increasing the length of time per bout sitting on the nest [22], [23]. Increased nest predation risk could also favour the evolution of shorter incubation periods to minimise the total time a nest is at risk [24], [25]. Bellbirds, therefore, might respond adaptively to the increased risk of predation by novel predators by lowering parental activity, increasing length of incubation on-bouts, and shortening incubation periods.

We examined behavioural and life history responses in the bellbird to changing nest predation risk on differing time scales. A few New Zealand offshore islands have never had exotic predators and provide a benchmark to compare with populations on the main New Zealand islands that have been exposed to novel predators beginning 700 years ago. As small islands might alter life history traits independently of predation risk (e.g. due to higher density), we also conducted a predator removal experiment on the mainland New Zealand to further examine whether bellbirds assess current predation risk and alter their behaviour and life history traits accordingly. Finally, to examine the effects of historical predation risk, we compared parental behaviour of bellbirds in New Zealand with two related honeyeaters (*Phylidonyris* spp.) in Tasmania, Australia. Tasmanian honeyeaters evolved with a variety of native mammalian predators, yet share a common ancestor with the bellbird. Thus, honeyeaters in Tasmania and New Zealand provide an opportunity to examine differences in responses to differing long-term exposure to mammalian predators.

## Methods

### Study areas

The bellbird is a medium-sized honeyeater (26–34 g) endemic to New Zealand [26]. The abundance and range of bellbirds has decreased since human settlement, but they survived within most native forest areas on the main islands of New Zealand and on several offshore islands [26]. Bellbirds were studied in three locations: (1) on Aorangi Island (35°28′ S, 174°44′ E), a forested island of approximately 66 ha, ∼22 km off the east coast of the North Island where exotic mammalian predators have never existed, (2) in Waiman Bush (42°20′ S, 173°40′ E), a 65 ha native forest located 15 km from Kaikoura where all exotic predators were continuously removed throughout the year from 2004 to 2007, and (3) in Kowhai Bush (42°22′ S, 173°36′ E), a 240 ha native forest located 10 km from Kaikoura, South Island where all exotic predators are present.

Parts of Aorangi Island were cultivated by Maori until 1820, but it was then abandoned and the island has remained uninhabited and declared a nature reserve in 1929. Polynesian rats were never introduced during Maori settlement on the island and to this day it remains free of all introduced predatory mammals. The island is far enough from the mainland that gene flow is likely to have been minimal, a possibility that is supported by the slightly different colouration of birds compared to the mainland [27]. The only potential predators present on the island are native and include Australasian harrier (*Circus approximans*), long-tailed cuckoo (*Eudynamys taitensis*), shining cuckoo (*Chrysococcys lucidus*) and perhaps the large Duvaucel's gecko (*Hoplodactylus duvaucelii*). In contrast, the mainland site at Kowhai Bush includes all species of exotic predators plus native avian predators. The birds in Kowhai Bush have co-existed with exotic mammalian predators for at least the last 700 years, a situation typical of that faced by all surviving native species on the main islands of New Zealand. Waiman Bush is at the same elevation and includes the same native forest habitat and avifauna as Kowhai Bush. The two sites are separated by about 5 km of mostly cleared agricultural land although connected by continuous forest at a higher elevation. Beginning in 2004, all species of exotic mammalian predators were removed from Waiman Bush using 38 tunnel traps to control mustelids, rats and hedgehogs, 8 Timms traps for possum and cat control, and 52 poison bait stations controlling rats and possums. A total of 90 stoats, 24 ferrets, 24 weasels, 23 possums, 137 rats, 218 hedgehogs, and 32 cats were caught in traps during this period and an additional unknown number of animals were killed by poison from the bait stations (or through secondary poisoning). It is not possible to permanently remove all predators from mainland sites but similar efforts to control predators at other New Zealand sites have lead to increased nest success and population increases of many native birds [28], [29], a general pattern that is evident on our study site as well [30].

While Aorangi Island is located further north than Kowhai and Waiman Bush (7° on a north south axis), all three sites are lowland coastal forests with a similar canopy structure and experience a similar maritime climate. The composition of the forest differs slightly between Aorangi and the two mainland sites and this is reflected in the nest sites selected by bellbirds. On Aorangi Island most bellbird nests are built in weeping matipo (*Myrsine divaricata*), the native vine *Muehlenbeckia* spp., and *Coprosma macrocarpa* [for details on vegetation see 31], [32], while in Kowhai and Waiman Bush bellbirds generally nest in kanuka (*Leptospermum ericiodes*) and in the shrub *Coprosoma robusta*. Nests at all three sites are placed so that they are well-concealed by dense vegetation and it is unlikely that any differences in parental behaviour between sites was due to differences in nest site placement.

We also studied honeyeaters in Tasmania, Australia to examine traits in a site where native predators have always existed. It is unknown which Australian honeyeater is phylogenetically the closest relative to the New Zealand bellbird [33], but we selected two species of native honeyeaters (crescent honeyeater *Phylidonyris pyrrhoptera* and New Holland honeyeater *P. novaehollandiae*) in Tasmania that are in the same family as bellbirds and so have a common ancestor. The crescent and New Holland honeyeaters are of similar size and morphology as the New Zealand bellbird, and the habitat preferences, life history traits, mating systems and parental behaviours are also similar among these three species. For example, mean clutch size is 2.8, 2.2 and 3.1 eggs in the crescent honeyeater, New Holland honeyeater and the bellbird, respectively; in all three species both the incubation and nestling period are 13–14 days long; all three species are socially monogamous and share parental care, whereby females are solely responsible for nest construction and incubation, but both sexes feed nestlings [26], [34]–[37]. The two Australian honeyeaters were studied in the Scamander Forest Reserve near St. Helens, Tasmania (41°27′ S, 148°15′ E). This is a 100 ha native forest block that is not subject to logging or hunting and contains a wide range of native predatory mammals and snakes. We chose to study honeyeaters in Tasmania because it is located at a similar latitude and experiences a similar maritime climate to our New Zealand study sites. Scamander Forest Reserve is also located at the same elevation as our New Zealand study sites and the forest structure is similar although it is dominated by gums (*Eucalyptus* spp.) with an acacia (*Acacia* spp.) and fern understorey. Like the bellbird, the open-cup nests of the Tasmanian honeyeaters were placed in the shrub and canopy layer and well-concealed by surrounding vegetation.

Data on life history and nesting behaviours were collected at all study sites from October until December each year (Aorangi: 2004–2005; Waiman Bush: 2004–2006; Kowhai Bush: 1998–2007; Tasmania: 2004–2005). Nests were found by following adults, and nests were monitored every 2–5 days to record nest success. Daily nest predation rates were calculated using the Mayfield method [38], [39]. We followed Hensler & Nichols [40] to calculate standard errors for Mayfield's daily predation probabilities, and analysed differences in daily predation rates using the CONTRAST programme [41]. Clutch size was determined for accessible nests. For nests found during nest-building or egg laying, we measured incubation periods as the period from last egg laid to the last egg hatched (to an accuracy of 2 days or less). To estimate parental visitation rates, we videotaped nests during both the incubation and the nestling stage using portable Sony Hi8 video cameras. Nests were filmed for the first 6 hours of the day, starting within 30 min of sunrise, except on Aorangi in 2004 when nests were taped later in the morning. Despite this difference in protocol, we pooled data across years because we did not detect significant differences on Aorangi between 2004 and 2005 in parental visits during incubation (*F*_1,17_ = 0.001, *p* = 0.98), incubation attentiveness (*F*_1,17_ = 3.6, *p* = 0.08), mean on-bout length (*F*_1,16_ = 0.14, *p* = 0.72), mean off-bout length (*F*_1,16_ = 3.05, *p* = 0.1), and nestling feeding visits (*F*_1,20_ = 2.86, *p* = 0.11) . Nests were filmed throughout the incubation period although we avoided filming nests within the first few days after laying. Incubation videos were scored for number of parental visits to the nest, nest attentiveness (measured as percentage of 6 h that females sat on the nest), and mean duration of incubation on and off-bouts [25], [42]. One incubation video from Aorangi was excluded because the female was extensively fed by her mate while on the nest, the only example of this behaviour we noted. As we expected higher visitation rates on the island with no exotic predators, the high rate of male visits to this nest increased our estimate of total visitation rate, such that the removal of this outlier makes our test of higher visitation rates in the absence of exotic predators more conservative. Nests with nestlings were videotaped within one day of nestlings breaking primary pinfeathers, to control for differences in developmental rates between locations or species and to quantify rates of parental visits to the nest to feed nestlings [9], [21].

Locating bellbird and honeyeater nests is time consuming, and given the high probability of nest failure (often before data on parental investment could be collected), multiple seasons across sites had to be sampled to increase sample sizes. To control for repeat sampling of females or pairs across seasons (no repeat sampling of females or pairs occurred within a season), we individually colour-banded 77 and 64 adult bellbirds on Aorangi Island in 2004 and in Kowhai Bush from 2000–2006, respectively. On Aorangi Island, nests of only one female were found in both seasons, and in Kowhai Bush nests of two females were found in two consecutive seasons. In all three cases banded females were paired with an unbanded male of unknown breeding history. To avoid repeat sampling of females, we randomly selected one incubation video and one nestling stage video per female for the analysis. The wide spatial distribution of filmed nests at all sites also minimised the chances of resampling unbanded birds more than once.

We tested whether life history traits and parental behaviours varied among locations by conducting ANOVAs after ensuring that the assumptions of an ANOVA were met (homoscedasticity and normality). We used LSD post-hoc tests to examine differences among individual sites when the ANOVA was significant. All means are reported±standard error.

## Results

**Figure 1:**
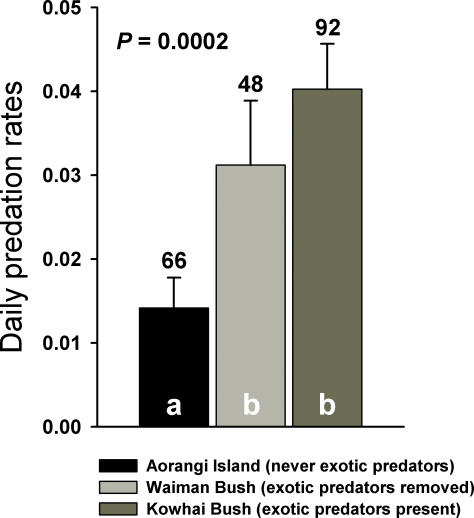
Daily nest predation rates for bellbirds on Aorangi Island, where exotic predators were never introduced, in Waiman Bush, where exotic predators were removed, and in Kowhai Bush, where all exotic predators are present. Shared letters within bars denote non-significant (i.e., *p*>0.05) statistical differences using the CONTRAST programme [41]. Figures above bars are the number of nests in each study site.

Daily nest predation rates for bellbirds were significantly lower on Aorangi Island (no exotic predators present) than in Waiman Bush (exotic predators removed) and Kowhai Bush (exotic predators present) (Figure 1). Daily predation rate was lower in Waiman Bush than in Kowhai Bush although the difference was not significant (Figure 1). Over a nesting cycle spanning about 30 days, these differences give a probability of a nest surviving to fledge of 65% for bellbirds on Aorangi, 39% for bellbirds in Waiman Bush and only 29% for bellbirds in Kowhai Bush.

**Figure 2:**
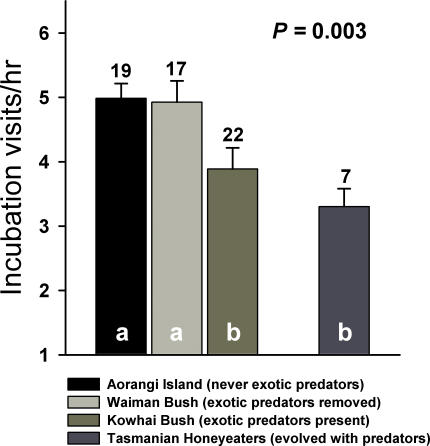
The number of parental visits to the nest per hour during incubation for bellbirds on Aorangi Island, where exotic predators were never introduced, for bellbirds in Waiman Bush, where exotic predators were removed, for bellbirds in Kowhai Bush, where all exotic predators are present, and for honeyeaters in Tasmania, which evolved with a range of native mammalian predators. Shared letters within bars denote non-significant (i.e., *p*>0.05) statistical differences based on LSD tests. Figures above bars are the number of nests filmed in each study site.

Parental behaviour varied among bellbird populations with the varying levels of predation risk. The number of parental visits to the nest during incubation varied significantly among the four sites (*F*_3,61_ = 5.12, *p* = 0.003, Figure 2). Bellbirds on Aorangi Island, where exotic predators were never introduced, and in Waiman Bush where predators were removed, visited their nests at similar rates. At both these sites bellbirds visited their nests more frequently than at Kowhai Bush, where a variety of exotic predators are present, and honeyeaters in Tasmania that evolved with native predators (Figure 2). We obtained the same results when we controlled for day of incubation and only included nests that were filmed during the middle of incubation (days 4–8 of a 14 day incubation period); parental visitation rates during the middle of incubation differed among sites (*F*_3,27_ = 5.59, *p* = 0.004) in a similar pattern: the rate on Aorangi was similar to the rate in Waiman Bush, which was higher than the visit rate in Kowhai Bush, which in turn was similar to that of honeyeaters in Tasmania.

Nest attentiveness (percent time females spend on the nest), in contrast to visit rates, did not differ among sites (*F*_3,61_ = 0.346, *p* = 0.8). Mean nest attentiveness by females was 67.8% (±1.59, *n* = 19) on Aorangi , 66.2% (±1.91, *n* = 17) in Waiman Bush, 68.5% (±1.73, *n* = 22) in Kowhai Bush, and 68.6% (±2.31, *n* = 7) in Tasmania. Thus bellbirds did not alter the total time spent incubating with increased predation risk but instead decreased the number of visits made to and from the nest by changing bout lengths.

**Figure 3:**
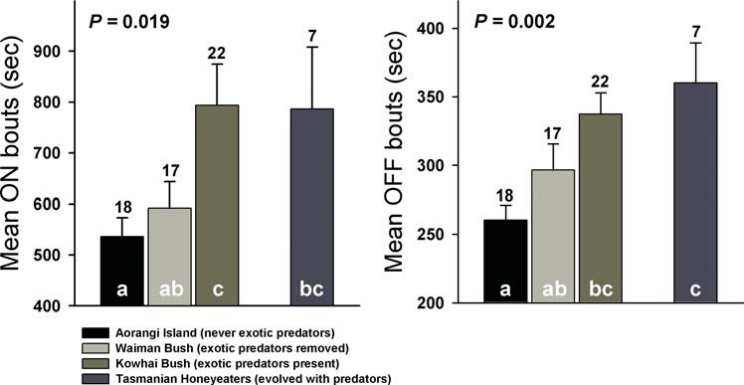
The mean time females spent on the nest per incubating bout (mean on-bouts) and the mean time females spent away from the nest (mean off-bouts) for bellbirds on Aorangi Island, where exotic predators were never introduced, for bellbirds in Waiman Bush, where exotic predators were removed, for bellbirds in Kowhai Bush, where all exotic predators are present, and for honeyeaters in Tasmania, which evolved with a range of native mammalian predators. Shared letters within bars denote non-significant (i.e., *p*>0.05) statistical differences based on LSD tests. Figures above bars are the number of nests filmed in each study site.

The mean time females spent on the nest per incubating bout differed among the four sites (*F*_3,60_ = 3.58, *p* = 0.019, Figure 3a). Duration of on-bouts during incubation were similar on Aorangi (no exotic predators present) and Waiman Bush (predators controlled), but both were shorter than in Kowhai Bush (no predator control; Figure 3a). Honeyeaters in Tasmania had similar durations of on-bouts as bellbirds in Kowhai Bush and Waiman Bush, but significantly longer than bellbirds on Aorangi (Figure 3a). Duration of off-bouts (time females spend away from the nest during each recess) also differed among sites (*F*_3,60_ = 5.75, *p* = 0.002) and mirrored the pattern observed in on-bouts with bellbirds showing increasing times spent away from the nest as predation risk increased: bellbirds had the shortest off-bouts on Aorangi and the longest in Kowhai Bush (Figure 3b). Honeyeaters in Tasmania had off-bouts similar to that observed in bellbirds in Kowhai Bush (Figure 3b).

**Figure 4:**
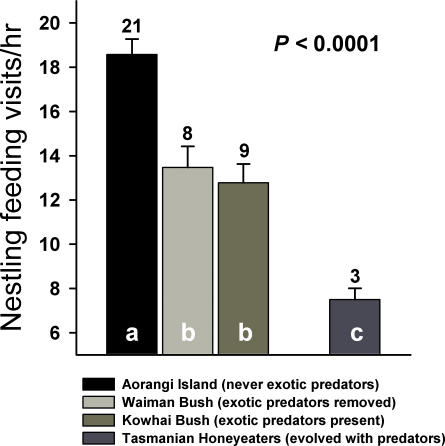
The number of parental visits to the nest per hour to feed nestlings for bellbirds on Aorangi Island, where exotic predators were never introduced, for bellbirds in Waiman Bush, where exotic predators were removed, for bellbirds in Kowhai Bush, where all exotic predators are present, and for honeyeaters in Tasmania, which evolved with a range of native mammalian predators. Shared letters within bars denote non-significant (i.e., *p*>0.05) statistical differences based on LSD tests. Figures above bars are the number of nests filmed in each study site.

Rate of parental feeding visits to the nest during the nestling period differed among all four sites (*F*_3,37_ = 19.274, *p*<0.0001) with bellbirds on Aorangi (no predators present) visiting nests more frequently than at either Waiman Bush (predators removed) or Kowhai Bush (predators present; Figure 4). There was no significant difference between Waiman and Kowhai Bush, so our predator removal experiment did not change the feeding behaviour of bellbirds. Parental feeding rates at both Waiman and Kowhai Bush were significantly higher than that observed in honeyeaters in Tasmania (Figure 4). When we controlled for brood size by only including nestling videos of 3-chick broods, nestling feeding rates still differed strongly among the sites (*F*_3,19_ = 9.623, *p*<0.0001), with the same significant differences among study sites.

In contrast to parental behaviour, bellbirds exhibited little change in other life history traits. Bellbird clutch size did not differ among the three sites in New Zealand (*F*_2,117_ = 0.251, *p* = 0.8). Mean clutch sizes were 3.14 eggs (±0.08, *n* = 57) on Aorangi (exotic predators never introduced), 3.08 eggs (±0.15, *n* = 12) in Waiman Bush (exotic predators removed experimentally), and 3.06 eggs (±0.09, *n* = 51) in Kowhai Bush (exotic predators present). The Tasmanian honeyeaters were not included in this analysis as sample sizes were small but all crescent honeyeaters laid 3 eggs (*n* = 6 nests) and all New Holland honeyeaters laid 2 eggs (*n* = 3).

Incubation period also did not differ among the three sites in New Zealand (*F*_2,22_ = 1.073, *p* = 0.4). Mean incubation periods were 14.1 days (±0.21, *n* = 11) on Aorangi, 14.6 days (±0.43, *n* = 7) in Waiman Bush, and 14.9 days (±0.60, *n* = 7) in Kowhai Bush. We were unable to gather enough information on incubation periods in Tasmanian honeyeaters, but published records indicate both crescent honeyeater (13.2±0.20 days, *n* = 5) [43] and New Holland honeyeater (13.4±0.12 days, *n* = 19) [43] have shorter incubation periods than the New Zealand bellbird.

## Discussion

We examined how an endemic island bird that evolved largely without terrestrial predators responds to the novel, and an unusually high, risk of predation due to the introduction of multiple, exotic, mammalian predators to New Zealand. We found that the presence of introduced mammalian predators in New Zealand over the past 700 years have induced shifts in parental behaviour in the endemic bellbird that appear to be adaptive. These changes converge on behaviours seen in other species of honeyeaters endemic to Australia, which co-evolved with a variety of predators, and which presumably evolved to minimise the risk from predation. Our results suggest that bellbirds, and perhaps other endemic island birds, are not stuck in an evolutionary “trap” as has been proposed, but instead have some capacity to adapt to novel changes in environment including that posed by the introduction of exotic predators.

Following a hypothesis by Skutch [19], parental activity at the nest can attract the attention of predators and increase nest predation risk [8], [9], [20]. An adaptive response would be to reduce activity with increasing predation risk [9], [23], [44], [45]. Our results suggest such adaptive responses in bellbirds: bellbirds on an offshore island without exotic predators and on the mainland predator- removal site had shorter on- and off-bouts that yielded higher parental visit rates than birds that have now co-existed with exotic predators for c. 700 years (Figures 1, 2). Moreover, bellbirds that coexist with exotic predators had long on- and off-bouts which reduced visit rates to a level similar to their Tasmanian relatives that evolved with mammalian predators (Figures 1, 2). Thus, our results suggest that New Zealand bellbirds are able to respond to exotic nest predation risk by altering their incubation behaviour in a manner similar to related species of honeyeater in Tasmania in a period not exceeding 700 years.

In contrast, historical differences appear to remain for parental activity during the nestling period. Bellbirds that co-exist with exotic predators decreased their nestling feeding rates compared to birds on the offshore island without predators, as expected under an adaptive shift, but did not increase feeding when we experimentally removed predators. Nevertheless, bellbirds on the mainland still fed their nestlings twice as often as honeyeaters in Tasmania suggesting the persistence of higher rates of nest visitation in the presence of exotic predators. Although we cannot rule out differences in diet between these species as a potential explanation, our results are also consistent with the view that bellbirds on the New Zealand mainland appear to be adapting to exotic predators but they retain behavioural traits present in naive populations.

Some life history traits appear to show little response to predation risk. Increased nest predation risk has been argued to favour decreased clutch size to reduce the number of nest visits that could attract the attention of predators and the overall period when the nest is vulnerable to nest predators [6], [19], [46]. Presentations of predator models have yielded clutch size reductions [47], [48], while a predator-removal experiment yielded no change in clutch size among eight coexisting passerine species [21]. Similar to the latter result, bellbirds did not reduce their clutch size and laid an average of 3 eggs (range of 2–4 eggs) at all sites despite the difference in predation risk among sites. Thus, this trait was less responsive than parental visitation rates to predation risk.

Female bellbirds increased the length of on- and off-bouts and thereby reduced parental activity, but did not increase their total incubation attentiveness with increased predation risk. Conway & Martin [23] found the same pattern across North American passerine species. Increased attentiveness can potentially shorten incubation and reduce nest predation risk [24], [25], but the lack of change in nest attentiveness is consistent with the lack of change in incubation periods that we observed. Increased attentiveness might compromise adult survival [24], [49]. Female bellbirds incubate alone, and males generally do not feed females during incubation (apart from the one exception noted in the Methods). As a result, females might require a set amount of time off the nest to replenish their resources to minimize mortality costs to themselves. Moreover even if females increased attentiveness, offspring might not have the physiological means to accelerate embryonic development. New Zealand songbirds typically have very long developmental periods that might reflect intrinsic mechanisms to enhance offspring quality and longevity that may not be altered by attentiveness. Nonetheless, the end result is that nest attentiveness and incubation periods did not change with nest predation risk and further demonstrates that traits differ in their responsiveness to nest predation risk.

Island species are often thought to lack the ability to adapt to novel predators, but our data suggest that at least some traits have shifted or are plastic in response to predation risk in ways that appear adaptive. Adaptive phenotypic evolution has recently received considerable attention in the literature, because it offers opportunities for new, innovative approaches to ecosystem management and conservation efforts [13], [50]–[53]. An eco-evolutionary perspective has been promoted [13], [50], [53], whereby contemporary evolution arising from the novel interaction between invasive and native species is considered. In practice, a study aimed at detecting the minimum thresholds of management required to induce the responses that allow the long-term persistence of native bird populations is now necessary to develop such a new management tool further. To this effect it would be useful to replicate our study using further removal experiments that varied in the extent to which predation risk is decreased, and to examine whether other island species have responded in a similar fashion as we found with bellbirds.

One of the main problems when attempting to measure contemporary evolution in native island birds in response to the introduction of exotic predators is that few island bird populations still exist in habitats that have not been affected by human-mediated changes. While changes in morphology and genetic variation can by studied by comparing current populations with museum collections [54], [55], the measurement of behavioural responses to introduced predators requires live bird populations in areas that remain relatively undisturbed by anthropogenic effects. Here, we had the opportunity to study such behavioural responses because of the unique situation in New Zealand where exotic predators were introduced, but a few offshore islands remained undisturbed. While we report a phenotypic change in parental behaviours of bellbirds, we are uncertain about the relative contributions of genetic and non-genetic effects. Regardless, we believe that an improved understanding of the adaptive potential of species facing drastic environmental change and the rate at which such threatened species can achieve phenotypic adaptation can aid future management efforts for the conservation of threatened island bird species.
